# Effects of Plant Coverage on the Abundance of Adult Mosquitos at an Urban Park

**DOI:** 10.3390/plants12050983

**Published:** 2023-02-21

**Authors:** Yunfeng Yang, David A. Ratkowsky, Jiaqi Yang, Peijian Shi

**Affiliations:** 1 College of Landscape Architecture, Nanjing Forestry University, 159 Longpan Rd., Nanjing 210037, China; 2 Tasmanian Institute of Agriculture, University of Tasmania, Private Bag 98, Hobart 7001, Australia; 3 Bamboo Research Institute, Nanjing Forestry University, 159 Longpan Rd., Nanjing 210037, China

**Keywords:** coverage of plants, generalized additive models, multiple linear regression, nonlinear effects, Xuanwu Lake Park

## Abstract

People who take a walk in urban parks, including or adjacent to a water body such as a river, a pond, or a lake, usually suffer from mosquito bites in summer and early autumn. The insects can negatively affect the health and mood of these visitors. Prior studies about the effects of landscape composition on the abundance of mosquitos have usually taken stepwise multiple linear regression protocols to look for the landscape variables that can significantly affect the abundance of mosquitos. However, those studies have largely overlooked the nonlinear effects of landscape plants on the abundance of mosquitos. In the present study, we compared the multiple linear regression (MLR) with the generalized additive model (GAM) based on the trapped mosquito abundance data obtained by using photo-catalytic CO_2_-baited lamps placed at the Xuanwu Lake Park, a representative subtropical urban scenic spot. We measured the coverage of trees, shrubs, forbs, proportion of hard paving, proportion of water body, and coverage of aquatic plants within a distance of 5 m from each lamp’s location. We found that MLR and GAM both detected the significant influences of the coverage of terrestrial plants on the abundance of mosquitos, but GAM provided a better fit to the observations by relaxing the limitation of the linear relationship hypothesis by MLR. The coverage of trees, shrubs, and forbs accounted for 55.2% of deviance, and the coverage of shrubs had the greatest contribution rate among the three predictors, accounting for 22.6% of the deviance. The addition of the interaction between the coverage of trees and that of shrubs largely enhanced the goodness of fit, and it increased the explained deviance of the GAM from 55.2% to 65.7%. The information in this work can be valuable for the planning and design of landscape plants to reduce the abundance of mosquitos at special urban scenic points.

## 1. Introduction

Adult mosquitos at urban scenic points, especially urban parks including or adjacent to some water bodies such as rivers, ponds, or lakes, pose a threat to visitors as vectors by biting them and spreading viruses [1]. Although the mosquitos of Chironomidae do not bite humans and their larvae can serve as food for fish and other aquatic insects [2,3], the adults can fly in flocks at a riverside or a lakeside and unfavorably influence the leisure and sports of visitors. Landscape composition and landscape heterogeneity can affect the species composition and abundance of mosquitos [1,4,5]. Among urban green landscapes, plants can significantly influence the diversity and abundance of adult mosquitos [6,7]. Only a few studies [5,7,8] have reported the effects of the coverage of landscape plants on the abundance of mosquitos trapped by chemically baited lamps. In addition to plant coverage, the species composition of plants also has an effect on the abundance of mosquitos from the evidence of the entomological and vegetation survey on residential properties based on the four-year records of service requests of residents concerning what is known about mosquitos, which was conducted in St. Johns County, Florida, USA [9]. Prior studies used simple correlation analysis between the abundance of mosquitos and each of the landscape variables [5,8], and a further study [7] used generalized linear mixed models on the principal components obtained from a principal components analysis of the landscape variables. It is well known that the two methods are based on the linear hypothesis between the explanatory variables (i.e., landscape variables or their principal components) and the response variable (i.e., the abundance of mosquitos) or its transform. However, in the real world, the effects of the explanatory variables on the response variable are seldom linear [10,11,12]. If landscape variables have nonlinear effects on the abundance of mosquitos, the above methods tend to fit less well and are even likely to lead to incorrect conclusions.

Generalized additive models [13,14,15] are suitable for detecting and explaining the nonlinear effects of explanatory variables on many types of response variable (e.g., presence–absence data, count data, etc.). Using partial residuals and observing the 1-D smooths of the explanatory variables can help reveal the nonlinear effects that the explanatory variables have. It has been widely applied in biological sciences, especially ecology [15,16]. In fact, there are many case studies that have shown the superiority of GAMs over linear models in the biological sciences (e.g., refs. [17,18,19]). Nevertheless, GAMs have been largely overlooked in prior studies that were related to the effects of landscape variables on mosquito abundance. In the present study, we tested whether the coverage of trees, shrubs, forbs, and other landscape variables significantly affect the abundance of mosquitos trapped by CO_2_-baited lamps placed in a subtropical urban park, and compared the GAM with the multiple linear regression model to test whether the former significantly improved the goodness of fit to the observed data.

## 2. Materials and Methods

### 2.1. Experiment

**Figure 1:**
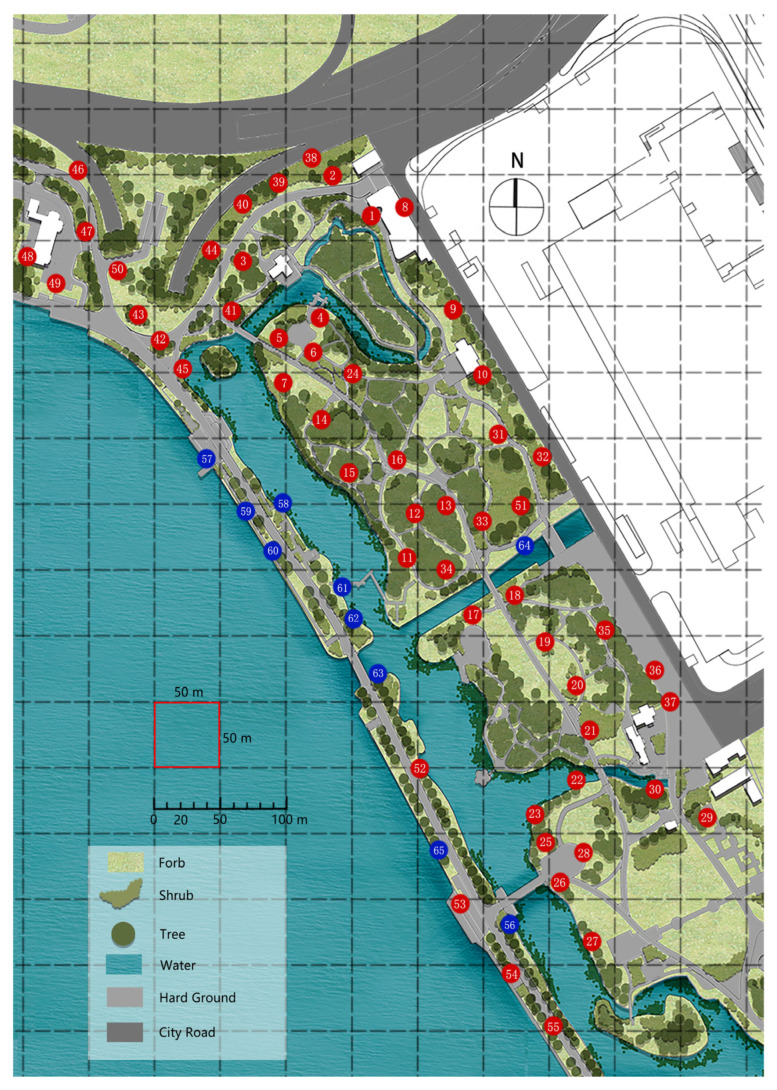
The study region and the site distribution of the CO_2_-baited lamps at Xuanwu Lake Park, Nanjing, China. Here, the red and blue points with numbers are the placement locations of lamps. The circle with a 5-m radius from an arbitrary blue point as the center included a certain proportion of water body. Blue circles are used whenever there is at least some amount of water in its 5-m radius, whereas there is no water within any of the red circles.

The study region is located at Xuanwu Lake Park in Nanjing, Jiangsu Province, China. We used CO_2_-baited lamps (Gongfuxiaoshuai LTS-M02 (24 W); Jixing Environmental Protection Technology Co., Ltd., Wuhan, China) at 65 sites distributed within the 550 m × 700 m study region (Figure 1). From 1 September to 14 September 2022 (with the exception of 13 September, which was a rainy day), the lamps were placed in the study region between 19:00 p.m. and 07:00 a.m. of the following morning. We counted the total abundance of mosquitos trapped in each lamp. Five species of mosquitos, viz. *Aedes albopictus* (Skuse), *Anopheles sinensis* Wiedemann, *Armigeres subalbatus* (Coquillett), *Culex tritaeniorhynchus* Giles, and *Culex pipiens* L. were found in the study region. Because we focus on the overall abundance of biting mosquitos at the park, we consider that there is no need to distinguish between species. In addition, there was a species of Chironomidae found in our experiment; however, it was neglected in the following analysis because it does not bite.

### 2.2. Landscape Variables

We calculated six landscape variables within a 5-m radius from the location where a lamp was placed: the coverage of trees (TC), the coverage of shrubs (SC), the coverage of forbs (FC), the proportion of hard paving (PHP), the proportion of water body (PWB), and the coverage of aquatic plants (APC). Table S1 in the online Supplementary Materials lists the information on census landscape plants. The proportion of hard paving denotes the proportion of building hard paving to the circular area (=25π m^2^), with each lamp’s location as the center. Plant coverage denotes the proportion of the vertical projected area to the circular area.

### 2.3. Statistical Models

We used stepwise regression for building the multiple linear regression models, adding or removing potential landscape variables in succession that significantly influenced the abundance of mosquitos (*y*). To guarantee that the predicted abundance was greater than zero, we used the natural logarithm of the abundance of mosquitos:

(1) $$\ln\left(y\right)=\beta_{0}+\beta_{1}\mathrm{TC}+\beta_{2}\mathrm{SC}+\beta_{3}\mathrm{FC}+\beta_{4}\mathrm{PHP}+\beta_{5}\mathrm{PWB}+\beta_{6}\mathrm{APC}$$

where $\beta_{0}$ to $\beta_{6}$ are parameters to be estimated. We used the “stepAIC” function in the “MASS” package [20] based on the statistical software R (version 4.2.0) [21] to find the optimum multiple linear model based on the lowest Akaike information criterion (AIC) that considers a trade-off between the model’s complexity and goodness of fit [22]. 

To test whether there are nonlinear effects of landscape variables on the abundance of mosquitos, we used the following generalized additive model (GAM) with the log link for the Poisson family:

(2) $$y\sim \alpha_{0}+s\left(\mathrm{TC}\right)+s\left(\mathrm{SC}\right)+s\left(\mathrm{FC}\right)$$

where $\alpha_{0}$ is the intercept, and *s*(·) represents a smooth function [14,15]. We did not consider other landscape variables in the GAM because those variables had been demonstrated to be insignificant by the multiple linear regression. We only checked (i) whether each of the three coverage variables had a significant influence on the abundance of mosquitos and (ii) how those three variables affected the abundance of mosquitos. The “gam” function in the “mgcv” package (version 1.8-40) was used to carry out the GAM fit [15]. 

To quantify the contribution rate (CR) of each plant coverage variable to the deviance of the GAM fit, we took the following approach [23]:

(3) $${\mathrm{CR}}_{i}=\frac{1/{\mathrm{DE}}_{i}}{\sum_{j=1}^{3}1/{\mathrm{DE}}_{j}}\times {\mathrm{DE}}_{0}\times 100\%$$

where DE*_i_* represents of the proportion of the deviance explained by a GAM dropping the *i*-th variable, and DE_0_ represents the proportion of deviance explained by the GAM using all three plant coverage variables simultaneously (i.e., TC, SC, and FC).

In general, for linear and nonlinear models, considering the interactions between or among explanatory variables can be beneficial to the improvement of the model’s goodness of fit. Because the mechanisms and forms of interactions cannot be known, the product between two or more variables is usually assumed to reflect such interactions [13]. However, whether an interaction term is worthwhile to add to a model requires two considerations: (i) whether there are implicit mechanisms (e.g., ecological, physiological, physical, chemical, or mental factors that can produce extra influences beyond the sum of the smooths of any two explanatory variables) that can result in an interaction, and (ii) whether the interaction item can largely improve the goodness of fit of a model. We added the product (TS) of TC and SC, the product (TF) of TC and FC, the product (SF) of SC and FC, and the product (TSF) of TC, SC, and FC to Equation (2) in different ways and thus, produced other eight GAMs: (i) *y* ~ s(TC) + s(SC) + s(FC) + s(TS) + s(TF) + s(SF), (ii) *y* ~ s(TC) + s(SC) + s(FC) + s(TF) + s(SF), (iii) *y* ~ s(TC) + s(SC) + s(FC) + s(TS) + s(SF), (iv) *y* ~ s(TC) + s(SC) + s(FC) + s(TS) + s(TF), (v) *y* ~ s(TC) + s(SC) + s(FC) + s(TS), (vi) *y* ~ s(TC) + s(SC) + s(FC) + s(TF), (vii) *y* ~ s(TC) + s(SC) + s(FC) + s(SF), and (viii) *y* ~ s(TC) + s(SC) + s(FC) + s(TSF), and for each with the log link for the Poisson family. When finding the target equation, we took a similar approach to Equation (3) to calculate the contribution rates to the deviance explained for all explanatory variables.

## 3. Results

Table 1 shows the results of the stepwise regression for the multiple linear models, with the maintained three landscape variables being the coverage of trees, shrubs, and forbs (i.e., TC, SC, and FC). However, SC and FC alone are not significant explanatory variables separately (*p* = 0.0907 and 0.1092, respectively, both being greater than 0.05). However, when we deleted SC and FC and only used TC as a single explanatory variable, the coefficient of determination (*r*^2^) decreased from 0.2193 to 0.1643. This indicates that SC and FC should not be removed from the multiple linear regression model. 

**Figure 2:**
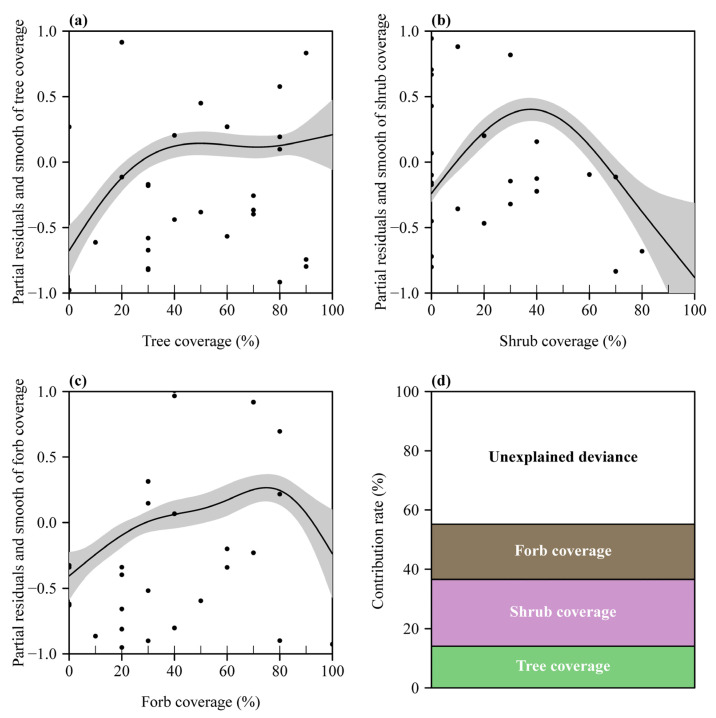
Partial residuals and smooth of each of the three plant coverage variables and the contribution rates to the deviance for the GAM fit. (**a**) Coverage of trees; (**b**) coverage of shrubs; (**c**) coverage of forbs; (**d**) the contribution rates of the three coverage to the deviance from the GAM fit based on the three landscape variables. In panels (**a**–**c**), the closed points represent the partial residuals; the solid curve represents the estimate of each 1-D smooth; and the gray area represents two standard errors above and below the estimate of the smooth. Partial residuals for a smooth term are the residuals that would be obtained by removing the term of interest from the model while leaving all other estimates fixed (i.e., the estimates for the term plus the residuals) [15]. Panel (**d**) shows the contribution rates of the three explanatory variables and the unexplained deviance by the GAM.

The results of fitting the GAM showed that the three explanatory variables (TC, SC, and FC) are all significant (Table 2), and they exhibited nonlinear effects on the total abundance of mosquitos that can be observed by the partial residuals and smooth of each predictor (Figure 2). When the coverage of trees was smaller than 40%, the abundance of mosquitos increased with the coverage of trees increasing; when it was greater than 40%, the abundance of mosquitos tended to be constant (Figure 2a). The effect of the coverage of shrubs tended to be an open downward parabola, and the maximum value corresponds to ca. 40% of the coverage of shrubs (Figure 2b). The abundance of mosquitos increased with the coverage of forbs increasing before the 80% forbs coverage and dropped after the 80% forbs coverage (Figure 2c). The three landscape plant coverage in total explained 55.2% of deviance of the GAM, among which the coverage of trees, shrubs, and forbs accounted for 14.1%, 22.6%, and 18.5% of deviance, respectively (Figure 2d). 

**Figure 3:**
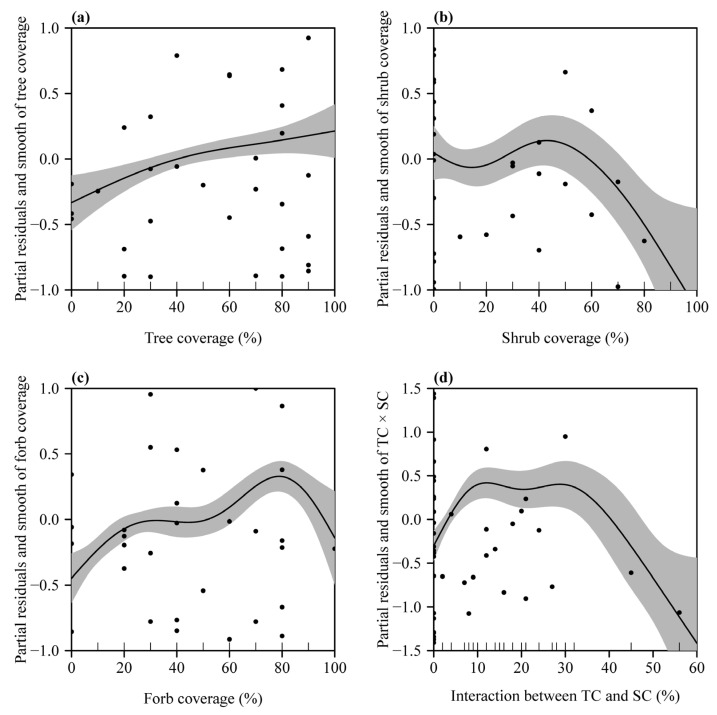
Partial residuals and smooth of each of the four plant coverage variables. (**a**) Coverage of trees; (**b**) coverage of shrubs; (**c**) coverage of forbs; (**d**) the interaction between the coverage of trees (TC) and that of shrubs (SC). In panels (**a**–**d**), the closed points represent the partial residuals; the solid curve represents the estimate of each 1-D smooth; and the gray area represents two standard errors above and below the estimate of the smooth.

**Figure 4:**
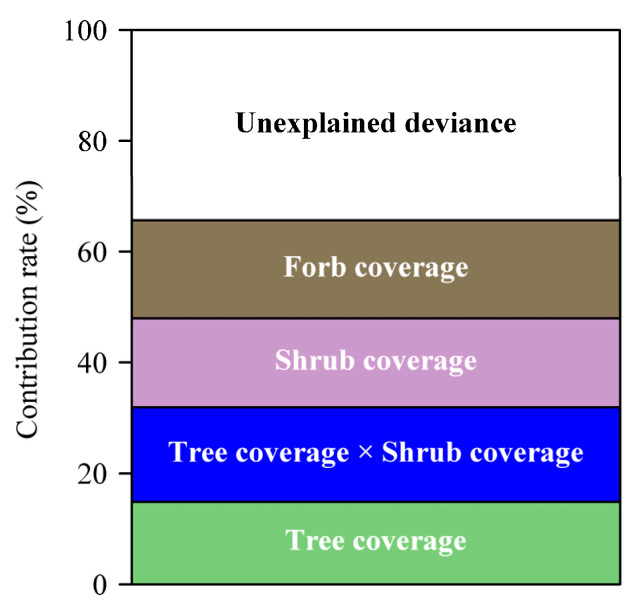
The panel shows the contribution rates of four explanatory variables (coverage of trees, shrubs, and forbs, plus the interaction between the coverage of trees and that of shrubs) to the deviance for the GAM.

By balancing the AIC and the significance for each of the explanatory variables, we found that the model comprising the interaction item TS was the best, with all explanatory variables significant (Table 3). This model increased the goodness of fit by 10.5% above the deviance of 55.2% explained by Equation (2) (Table 3). We checked the partial residuals and smooth of the explanatory variables and found that the effects of TC, SC, and FC changed to some degree because of the addition of the interaction item TS (Figure 3a–c). Comparing Figure 2 and Figure 3, it seems that the graphs for trees and forbs (i.e., parts (a) and (c)) are rather similar in the two figures, but that for shrubs, in the region of 0% to 30% (approx.), there is a distinct difference, with the graph for TS included being more or less the sum of the shrub coverage and interaction smooths. Relatively speaking, the interaction item TS has a larger effect on the abundance of mosquitos between a predictor’s range of 0% and 40% than those outside this range (Figure 3d). The four explanatory variables in total explained 65.7% of the deviance of the GAM, among which the interaction item TS accounted for 17.7% of the deviance (Figure 4).

## 4. Discussion

### 4.1. Why Did the Multiple Linear Model Explain a Lower Proportion of Deviance?

Relative to the 55.2% explainable deviance by the GAM, the multiple linear model only accounted for 21.9% of the deviance when using the same three plant coverage variables as explanatory variables, which did not even reach half of the goodness of fit for the former. This further confirms that in the real world, most effects of predictor variables on the response variable tend to be nonlinear [10,11,12]. If one attempts to use a linear relationship hypothesis between the predictor(s) and the response variable (or its transform) to fit the data, an incorrect conclusion may be drawn. The coverage of shrubs and forbs in the multiple linear model was not demonstrated to be significant in affecting the abundance of mosquitos because each of these two landscape variables affected the response variable parabolically (Figure 2b,c). Such parabolic effects are likely to result in the erroneous conclusion that the fitted regression line tends to be parallel to the *x*-axis if we are restricted to using only linear terms [24]. The use of the simple linear relationship goes against the fact of a nonlinear effect of SC and FC on the abundance of mosquitos and, therefore, leads to a worse goodness of fit relative to the GAM fit. This supports the finding that, in ecological research, the generalized additive (mixed) models, embodying the philosophy of “let the data speak”, have been demonstrated to be better than the linear (mixed) models when the effects of the explanatory variables are unknown [15,19,25,26,27]. At the very least, the residuals and smooth of each explanatory variable can provide a clue to choosing an appropriate linear or nonlinear mathematical expression for that variable. 

### 4.2. Interactions between Any Two Plant Coverage Variables

In the present work, the interactions between any two plant coverage variables were all hypothesized to be in the form of a product. We must admit that this multiplication hypothesis of interactions is somewhat subjective and can be potentially improved by other mathematical forms when the mechanisms of interactions can be clearly known. However, it is beyond the scope of the present work. The multiplication hypothesis of interactions has been widely used in multiple linear models and GAMs (see the discussion in ref. [13]). In fact, the multiplication hypothesis is also used to explain the complex dynamics caused by two or more factors with unknown mechanisms and forms of interactions in other study areas (e.g., refs. [28,29]). The addition of the interaction (TS) between the coverage of trees and that of shrubs largely enhanced the goodness of fit, and it increased the explained deviance of the GAM from 55.2% to 65.7%. Nevertheless, there does not seem to be any simple explanation for the improvement for the goodness of fit due to the addition of TS. We cannot exclude the possibility of over-fitting caused by this interaction item, which frequently occurs for the addition of an interaction to a model [30]. Whether it results from over-fitting deserves future investigation and requires more relevant experimental evidence to confirm or refute it. 

## 5. Conclusions

We explored the influence of six landscape variables on the abundance of mosquitos trapped by CO_2_-baited lamps at 65 sites at Xuanwu Lake Park, a representative subtropical urban park in which we found a total of five species of biting mosquitos. We found that the coverage of trees, shrubs, and forbs within a 5-m radius of each lamp’s location significantly affected the abundance of mosquitos. We compared the multiple linear regression model with the generalized additive model (GAM), and the latter had better goodness of fit and accounted for 55.2% of the deviance. Among the three explanatory variables (i.e., the coverage of trees, shrubs, and forbs), the coverage of shrubs made the largest contribution and explained 22.6% of the deviance. We analyzed the reason that the multiple linear regression model had a low coefficient of determination, which was caused by the incorrect hypothesis of the linear relationship between the predicator(s) and the response variable, and recommended using the GAMs in future relevant studies to explore the nonlinear effects of landscape variables on the abundance of mosquitos. In addition, we also discuss the influences of the interactions between any two plant coverage variables on the response variable but suggest using the interactions of the explanatory variables with caution, given that the mechanisms of the interactions are unknown.

## Figures and Tables

**Table 1:** Results of the stepwise regression for the multiple linear models.

Formula	AIC
ln(*y*) ~ TC + SC + FC + PHP + PWB + APC	−72.66
ln(*y*) ~ TC + SC + FC + PHP + PWB	−74.66
ln(*y*) ~ TC + SC + FC + PHP	−76.54
ln(*y*) ~ TC + SC + FC	−78.17

Here, TC represents the coverage of trees; SC represents the coverage of shrubs; FC represents the coverage of forbs; PHP represents the proportion of hard paving; PWB represents the proportion of water body; APC represents the coverage of aquatic plants; *y* represents the total abundance of mosquitos; AIC is Akaike information criterion.

**Table 2:** Approximate significance of smooth terms.

Smooth Terms	df	χ^2^	*p*-Value
*s*(TC)	3.31	53.94	<0.001
*s*(SC)	2.94	108.51	<0.001
*s*(FC)	4.30	39.70	<0.001

Here, TC, SC, and FC represents the coverage of trees, shrubs, and forbs, respectively; *s*(·) represents the smooth of the coverage’s partial residuals; df is the reference degrees of freedom which need not be an integer [14,15].

**Table 3:** Comparison among different candidate generalized additive models.

Formula	AIC	Deviance Explained	Insignificant Items
*y* ~ s(TC) + s(SC) + s(FC)	538.99	55.2%	−
*y* ~ s(TC) + s(SC) + s(FC) + s(TS) + s(TF) + s(SF)	483.26	72.6%	s(TC) and s(SF)
*y* ~ s(TC) + s(SC) + s(FC) + s(TF) + s(SF)	501.82	65.5%	−
*y* ~ s(TC) + s(SC) + s(FC) + s(TS) + s(SF)	501.11	65.7%	s(SF)
*y* ~ s(TC) + s(SC) + s(FC) + s(TS) + s(TF)	485.23	70.5%	s(TC)
*y* ~ s(TC) + s(SC) + s(FC) + s(TS)	499.48	65.7%	−
*y* ~ s(TC) + s(SC) + s(FC) + s(TF)	523.60	59.6%	−
*y* ~ s(TC) + s(SC) + s(FC) + s(SF)	523.25	59.7%	−
*y* ~ s(TC) + s(SC) + s(FC) + s(TSF)	509.67	63.4%	−

Here, each formula used the log link for the response variable; TC, SC, and FC represents the coverage of trees, shrubs, and forbs, respectively; TS represents TC × SC; TF represents TC × FC; SF represents SC × FC; TSF represents TC × SC × FC. “Insignificant items” denotes the smooth terms that are not significant at the 0.05 significance level, and “−” represents that there was no insignificant item in the corresponding model.

## Data Availability

The data used in the present work can be obtained from Yang and Yang (2023).

## Supplementary Materials

The following supporting information can be downloaded at: https://www.mdpi.com/article/10.3390/plants12050983/s1, Table S1: Information of census landscape plants.
